# Exercise‐based cardiac rehabilitation is associated with a normalization of the heart rate performance curve deflection

**DOI:** 10.1111/sms.13462

**Published:** 2019-05-28

**Authors:** Stefan Heber, Marina Sallaberger‐Lehner, Maria Hausharter, Ivo Volf, Helmuth Ocenasek, Harald Gabriel, Rochus Pokan

**Affiliations:** ^1^ Institute of Sport Science, Center for Sport Science and University Sports University of Vienna Vienna Austria; ^2^ Institute for Physiology, Center for Physiology and Pharmacology Medical University of Vienna Vienna Austria; ^3^ CARDIOMED Centre for Outpatient Cardiac Rehabilitation Linz Austria; ^4^ Department of Medicine II, Division of Cardiology Medical University of Vienna Vienna Austria

**Keywords:** cardiac rehabilitation, cardiorespiratory fitness, coronary heart disease, exercise test, intensity prescription, lactate turn point

## Abstract

The heart rate (HR) rises with increased power output, whereby in most healthy individuals, the slope of HR levels off with higher intensity. This corresponds to a downward deflection of the heart rate performance curve (HRPC). Conversely, in patients after myocardial infarction, an upward HRPC deflection is frequently observed that is especially pronounced in patients with compromised left ventricular ejection fraction. To investigate whether regular endurance training during cardiac rehabilitation might normalize HRPC, data of 128 male patients were analyzed. All patients performed three exercise tests: at baseline, after 6 weeks, and after 1 year. Ninety‐six patients exercised regularly according to guidelines for 1 year (training group, TG), and 32 stopped after 6 weeks (control group, CG). Similarly, upward‐deflected HRPCs were observed at baseline and after 6 weeks in both groups. After 1 year, TG patients had less upward‐deflected HRPCs compared with CG ones, corresponding to a partial normalization. Greater changes in HRPC deflection were associated with larger improvements in cardiorespiratory fitness. Our results might indicate improved myocardial function due to long‐term rehabilitation. Further, HRPC alterations over time should be considered when prescribing exercise intensities using a target HR, as deflection flattening might render the intensity of corresponding exercise insufficient.

## 1 INTRODUCTION

### 1.1 Background

Exercise training represents a cornerstone of cardiac rehabilitation programs for patients with coronary heart disease.^1^ It reduces cardiovascular mortality^2^ and contributes to normalization of impaired myocardial contractile function after myocardial damage,^3^ indicated by improved left ventricular ejection fraction (LVEF).^4^ Additionally, it improves cardiorespiratory fitness^5^ and has favorable effects in a multitude of chronic diseases.^6^ Recently, it was also shown that cardiac rehabilitation reduces the economic burden caused by cardiovascular disease.^7^

To maximize favorable effects of exercise training in cardiac rehabilitation, training guidelines also specify exercise intensity among other parameters.^1^ Most guidelines recommend progression from moderate to vigorous‐intensity aerobic endurance exercise.^8^ Monitoring of exercise intensity is essential, as insufficient intensities might be ineffective in eliciting the desired training effects,^9^ while extremely high intensities might represent a risk for patients.^10^ For this purpose, heart rate monitoring is suggested as an option by guidelines of most major cardiac rehabilitation societies.^11^ Compared to other means of intensity monitoring, as for example measurement of power output or oxygen uptake, heart rate monitoring is technically less demanding and can be undertaken by a substantial proportion of patients independently.

To determine an adequate target heart rate for exercise training, a heart rate performance curve (HRPC) determined in an incremental exercise test can be used, plotting the heart rate against the power output in watts. In most healthy young individuals, the HRPC is S‐shaped with a downward deflection beyond a certain level of intensity. Sometimes, linear or upward‐deflected HRPCs can be observed.^12^, ^13^ Specifically, in a study investigating 227 healthy young male volunteers, linear or upward‐deflected HRPCs were only observed in 6% and 8% of subjects, respectively.^14^

Interestingly, the degree of HRPC deflection is associated with changes in LVEF during exercise tests. Individuals with a linear HRPC show a smaller LVEF upon exhaustion after an exercise test compared to those with a downward deflection.^15^ In line with this, the reduction in LVEF observed during the last minutes of an incremental exercise test was inversely correlated with the HRPC downward deflection.^16^ Notably, the HRPC of patients who survived a myocardial infarction is frequently characterized by an upward deflection, and individuals with a more pronounced upward deflection show a more pronounced reduction of LVEF.^17^ The pathophysiological value of the HRPC deflection is also supported by more atypical forms in type I diabetic patients with higher HbA1c values.^18^

HRPC patterns have important implications for heart rate‐based exercise intensity prescriptions.^19^ In case of a linear HRPC, the percentage of maximal power output that serves as target intensity roughly equals the percentage of HR (heart rate) reserve at that intensity. However, in the case of an upward deflection, prescribing a particular percentage of HR reserve as target HR results in a higher percentage maximal power output, representing a possibly dangerous level of training intensity. Conversely, patients with a downward deflection would undertake insufficient levels of training intensity when reaching the prescribed heart rate.

Based on clinical experience, we expected that the degree of HRPC deflection would change throughout exercise‐based cardiac rehabilitation. Specifically, we assumed that the upward deflection usually observed in patients with coronary heart disease will reform, resulting in a linear or even downward‐deflected HRPC as usually observed in healthy individuals.

Cardiac rehabilitation is generally scheduled into three phases. Phase I corresponds to early in‐hospital mobilization after an acute event, with the aim to prevent complications secondary to immobilization. Phase II includes supervised training sessions. During this period, patients should clinically stabilize, and the foundation for favorable long‐term lifestyle changes should be laid. This includes physical activity counseling and the prescription of exercise training, nutritional counseling, as well as management of traditional risk factors for cardiovascular disease, as smoking cessation, blood pressure, and lipids. Phase II can generally be performed in an in‐ and outpatient setting, whereby the latter applies to this study. Phase III is performed in an outpatient setting and aims to provide sustained delivery of preventive and rehabilitative services. It consists both of supervised and independently performed training sessions throughout 1 year.^1^

### 1.2 Objectives

The primary objective of the study was to investigate whether exercise‐based cardiac rehabilitation normalizes HRPC deflection. Second, we aimed to evaluate whether changes in cardiorespiratory fitness are associated with changes in the HRPC deflection.

## 2 MATERIALS AND METHODS

### 2.1 Study design and setting

This retrospective, observational cohort study was approved by the local ethics committee of the Medical University of Vienna (Vote No. 1931/2015) and was conducted in accordance with the World Medical Association Declaration of Helsinki. Reporting follows the STROBE‐guidelines^20^ for observational studies.

**Figure 1:**
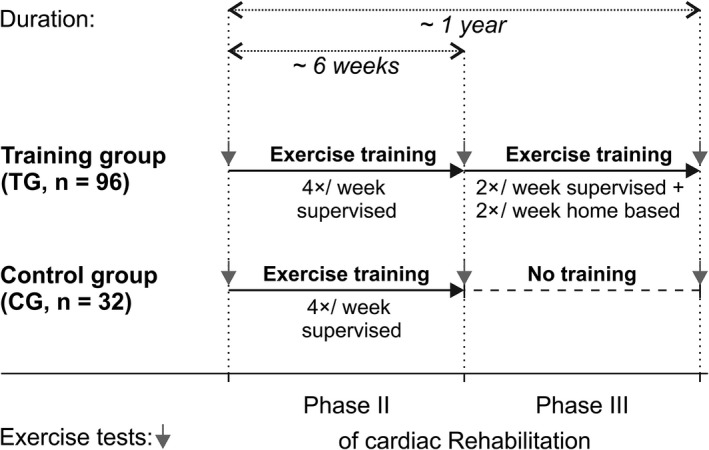
Overview of the retrospective study design. Patients who exercised regularly during phase II and phase III of cardiac rehabilitation were defined as training group, and patients who stopped after phase II were served as control group. Gray arrows pointing downwards indicate incremental exercise tests with the measurement of cardiorespiratory fitness and the degree of heart rate performance curve deflection (*K*_HR_)

Data of 128 male patients who have undergone outpatient cardiac rehabilitation between April 2010 and July 2015 at the CARDIOMED Centre for outpatient cardiac rehabilitation, Linz, AUSTRIA, were evaluated. For all participants, data of three time points were analyzed: (a) at the beginning of phase II cardiac rehabilitation, (b) approximately 6 weeks later, reflecting the transition from phase II to phase III, and (c) after approximately 1 year, corresponding to the end of phase III cardiac rehabilitation. Patients were subdivided into two groups: patients who exercised regularly according to guidelines^21^ throughout the whole observation period (training group, TG) and those who quit exercise training after phase II (control group, CG). During phase II (6 weeks), all training sessions were supervised and were performed in an outpatient setting. During phase III, two training sessions per week were supervised, and the patients were instructed to perform two additional training sessions independently at home (Figure 1). Under the assumption that exercise training affects HRPC deflection, we tested two main hypotheses: First, throughout phase II we hypothesized no difference in the change of HRPC deflection between the two groups. Second, throughout phase III we hypothesized to observe a different change in HRPC deflection over time between the two groups.

#### 2.1.1 Exercise training

Exercise training was performed according to the Austrian guidelines for cardiac rehabilitation^21^ and consisted of endurance exercise on bicycle ergometers. Intensity corresponded to 90% of the power output achieved at LTP2. The duration of each training session was 45 minutes, including 5 minutes warm up and 10 minutes cool down with an intensity corresponding to 30% of maximal power output. Exercise training is described in detail in the Appendix S1.

### 2.2 Participants

Generally, patients are enrolled in the outpatient cardiac rehabilitation program according to indications and contraindications for outpatient cardiac rehabilitation.^21^ These criteria also represent inclusion and exclusion criteria for this study and encompass, for example, status post‐myocardial infarction, stable coronary heart disease, or status post‐percutaneous coronary intervention. Only male patients who exercised regularly under supervision during phase II cardiac rehabilitation were included.

Patients were admitted according to pre‐existing diagnoses. Diagnoses were made by specialists for internal medicine/cardiology, who referred the patients to the rehabilitation center. Newly developed diseases were diagnosed by the medical directors of the cardiac rehabilitation center CARDIOMED Linz, including two specialists for internal medicine, and a general practitioner.

### 2.3 Variables

#### 2.3.1 Primary outcome measure: degree and direction of the HRPC deflection (*K*_HR_)

In accordance with previous studies, *K*_HR_ was used to assess the degree and direction of the HRPC deflection.^14^, ^17^, ^19^, ^22^, ^23^, ^24^ A *K*_HR_ value of 0 indicates a HRPC with constant slope; a negative *K*_HR_ value indicates a decrease and a positive *K*_HR_ value an increase in the slope toward the end of the exercise test. Estimation of *K*_HR_ is further described in the Appendix S1.

#### 2.3.2 Secondary outcome measures: parameters of cardiorespiratory fitness

Parameters of cardiorespiratory fitness as maximal power output and power output at lactate turn points 1 and 2 (LTP1 and LTP2) were assessed during the incremental exercise tests that were performed according to the Austrian guidelines for ergometry^25^ and are described in the Appendix S1. LTP1 and LTP2 were determined by linear regression breakpoint analysis as described.^22^, ^26^

#### 2.3.3 Primary predictors for hypothesis testing

The between‐subjects factor “group” consisted of the levels “training group” and “control group.” The within‐subjects factor “time” consisted of the levels “baseline,” “6 weeks,” and “one year.”

#### 2.3.4 Potential confounders and effect modifiers

The patient's age in years, their baseline body weight, their baseline power output in watts, their smoking status (yes/no), and the use of β‐blockers (yes/no) were included in the models for adjustment. Models with W/kg body weight as dependent variable were only adjusted for age, smoking status, and β‐blocker intake.

### 2.4 Data sources/management

*K*_HR_, LTPs, and power output were determined based on data acquired during incremental exercise tests by a person blinded to group membership of patients.

### 2.5 Study size

The sample size was chosen to detect a group difference of 0.15 *K*_HR_ units at the end of phase III cardiac rehabilitation with a power of 0.9, accepting a type I error rate of 0.05. Estimation of the necessary sample was performed with a sample size calculator for independent samples *t* tests and was based on the assumption of a pooled SD of 0.25 *K*_HR_ units. Based on previous experience in the outpatient cardiac rehabilitation center, we expected approximately every fourth patient to stop exercise training after phase II. Consequently, we chose a group size ratio of 3:1 for estimating sample size, resulting in 96 patients for the training group and 32 patients for the control group.

### 2.6 Quantitative variables

Continuous predictors as age or BMI were added to the statistical model in their original form and were not categorized.

### 2.7 Statistical methods

The primary statistical analysis was performed using a mixed linear model to test a “group” by “time” interaction, indicating that the K_HR_ change over time was different between groups. Importantly, the potential confounders’ age, baseline weight, baseline power output, and the proportion of patients taking β‐blockers were included in the model as covariates. In this way, the main results can be interpreted as if (a) all patients had exactly the same age, (b) all patients had exactly the same body weight and power output at baseline, and (c) the proportion of patients taking β‐blockers was the same at baseline and remained constant over time. Analogous models were used for secondary outcome variables. Relationships between changes in power output and changes in *K*_HR_ were tested using regression analyses. *P*‐values ≤0.05 were considered significant (*P ≤ 0.05; **P ≤ 0.01; ***P ≤ 0.001). All reported *P*‐values are results of two‐tailed tests. Statistical analysis was performed with IBM SPSS Statistics 24; graphs were created with GraphPad Prism 6. A detailed description of statistical analyses is provided in the Appendix S1.

## 3 RESULTS

### 3.1 Participants

Of 128 patients, 96 patients regularly exercised throughout phase II and phase III (training group, TG), and 32 stopped participating in regular exercise training sessions after phase II cardiac rehabilitation (control group, CG). Baseline characteristics of both groups are presented in Table 1. There were no significant baseline differences between groups after adjustment for multiplicity.

**Table 1:** Baseline patient characteristics

Characteristic	Unit		Group		Group difference *P*	
			Training (n = 96)	Control (n = 32)	Crude	Adjusted (Bonf.‐Holm)
Age	y	Mean ± SD	56.5 ± 9.1	60.2 ± 9.8	0.054	1
BMI	kg/m^2^	Median (IQR)	28.2 (25.6‐30.5)	27.4 (25.1‐30.1)	0.44	1
Resting heart rate	bpm	Mean ± SD	70.2 ± 12.8	67.0 ± 10.6	0.21	1
Maximal heart rate	bpm	Mean ± SD	137.1 ± 19.9	131.5 ± 22	0.18	1
Maximal power output	W/kg	Mean ± SD	1.96 ± 0.53	1.85 ± 0.45	0.28	1
Degree of heart rate deflection (*K*_HR_)		Mean ± SD	0.22 ± 0.29	0.27 ± 0.18	0.31	1
Current β‐blocker use		n (%)	65 (67.7)	24 (75)	0.44	1
Current low‐dose aspirin (acetylsalicylic acid) intake		n (%)	86 (89.6)	31 (96.9)	0.12	1
Current ADP receptor antagonist intake		n (%)	23 (24)	15 (46.9)	**0.01**	0.32
Current statin use		n (%)	89 (92.7)	27 (84.4)	0.46	1
Statin myopathy		n (%)	6 (6.3)	2 (6.3)	**1**	1
ACE inhibitor		n (%)	69 (71.9)	20 (64.5)	0.43	1
Current smoker		n (%)	40 (41.7)	5 (15.6)	**0.01**	0.32
Coronary heart disease		n (%)	82 (85.4)	28 (87.5)	0.77	1
St.p. myocardial infarction		n (%)	45 (46.9)	11 (34.4)	0.21	1
Ischemic cardiomyopathy		n (%)	5 (5.2)	2 (6.3)	1	1
Non‐ischemic cardiomyopathy		n (%)	1 (1.0)	0 (0)	1	1
Hypertensive cardiomyopathy		n (%)	2 (2.1)	3 (9.4)	0.1	1
Hypertrophic cardiomyopathy		n (%)	2 (2.1)	0 (0)	1	1
St.p. percutaneous coronary intervention		n (%)	32 (33.3)	7 (21.9)	0.22	1
St.p. stent implantation		n (%)	72 (75)	20 (62.5)	0.17	1
St.p. bypass grafting		n (%)	5 (5.2)	3 (9.4)	0.41	1
Implanted pacemaker^a^		n (%)	2 (2.1)	1 (3.1)	1	1
Implanted defibrillator		n (%)	5 (5.2)	2 (6.3)	1	1
Cerebrovascular disease		n (%)	6 (6.3)	4 (12.5)	0.27	1
Peripheral artery disease		n (%)	4 (4.2)	2 (6.3)	0.64	1
Chronic obstructive pulmonary disease		n (%)	4 (4.2)	5 (15.6)	**0.04**	1
St.p. pulmonary embolism		n (%)	1 (1.0)	2 (6.3)	0.15	1
Type II Diabetes mellitus		n (%)	22 (22.9)	3 (9.4)	0.12	1
Hyperlipidemia		n (%)	80 (83.3)	27 (84.4)	0.89	1
Hypertension		n (%)	65 (67.7)	21 (65.6)	0.83	1
Hyperuricemia		n (%)	10 (10.4)	1 (3.2)	0.29	1

Abbreviations: Bonf.‐Holm, Bonferroni‐Holm; bpm, beats per minute; SD, standard deviation; St.p., status post.

*P* < 0.05 (in bold).

a Pacemaker was not active during exercise tests.

### 3.2 Main results

#### 3.2.1 Effects of exercise training on HRPC deflection

**Figure 2:**
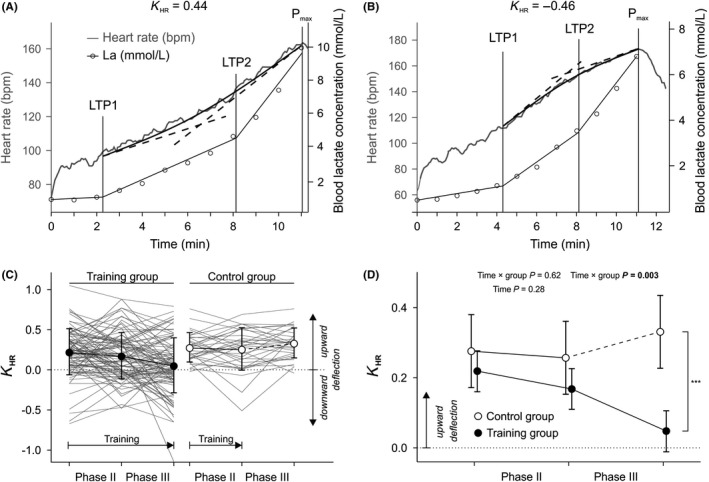
Effects of exercise training during phase II and phase III cardiac rehabilitation on heart rate performance curve (HRPC) deflection (*K*_HR_). A and B, Exemplary HRPCs. Time indicates the duration of an incremental exercise test. Blood lactate concentration after each step is used to determine LTP1 and LTP2. The region between LTP1 and the end of the exercise test (max) is used to determine *K*_HR_ by fitting a quadratic function to the heart rate data and relating the slopes of tangents at LTP2 and max (dotted lines) to each other (A) Upward‐deflected HRPC indicated by positive *K*_HR_. B, Downward‐deflected HRPC indicated by negative *K*_HR_. C, Descriptive statistics. *K*_HR_ values of each patient of the training group (n = 96) and the control group (n = 32) shown by thin, gray lines. Symbols indicate group means, and error bars show standard deviations. Horizontal arrows indicate the period in which regular exercise training was performed in each group. D, Inferential statistics. Estimated marginal *K*_HR_ value means of both groups with 95% confidence intervals after adjustment for the potential confounders age, baseline body weight, baseline power output in watts, smoking status (yes/no), and the use of β‐blockers (yes/no). The model is also adjusted for changes in β‐blocker intake over time. Symbols of each time point are slightly separated in *x*‐axis direction to avoid overlapping error bars. Note the adjusted *y*‐axis scaling compared to A. ***P < 0.0001 and the vertical bracket indicate the group difference at the end of phase III rehabilitation

Exemplary up‐ and downward‐deflected HRPCs with respective *K*_HR_ values are presented in Figure 2A,B. Individual changes in *K*_HR_ values over time for both groups are shown together with means and SD for each group and time point (Figure 2C). Age, baseline power output, body weight, and the number of individuals taking β‐blockers at each time point were considered potential confounders. Confounder‐adjusted estimated marginal means of *K*_HR_ values with 95% confidence intervals for each time point for each group are depicted in Figure 2D. Notably, at baseline, estimated *K*_HR_ value means of both groups were >0 and the 95% confidence intervals did not include 0, indicating a significant upward deflection in both groups at baseline.

The *K*_HR_ value change over time was generally different between groups (time × group interaction *P* < 0.001). Subsequent analyses showed that this was not the case in phase II, but in phase III (time × group interactions *P* = 0.62 and *P* = 0.003). Further, there was no change in *K*_HR_ during phase II in both groups (main effect time *P* = 0.28). Contrasts showed that groups did not differ concerning their mean *K*_HR_ values at the beginning of phase III, but at the end (*P* < 0.001). The 95% confidence interval of the TG at the end of phase III included 0 (dotted horizontal line), indicating that, in contrast to all other time points, there was no significant upward deflection in this group at this time point.

To address the question whether effects differ between patients taking β‐blocker at baseline and those who do not, this variable was included as an additional factor in another analysis, which showed no effects of baseline β‐blocker intake (Appendix S1A, time × group × β‐blocker interaction and main effect of β‐blocker *P* = 0.71 and *P* = 0.69). Analogous analyses were performed for ADP receptor antagonists, statins, and ACE inhibitors. There was no evidence of confounding by these drugs (data not shown). Additionally, confounding by type 2 diabetes was statistically tested. Although there was no evidence of confounding (time × group × type 2 diabetes interaction and main effect of type 2 diabetes interaction *P* = 0.21 and *P* = 0.31), substantial mean differences were observed.

#### 3.2.2 Effects of exercise training on parameters of cardiorespiratory fitness

**Figure 3:**
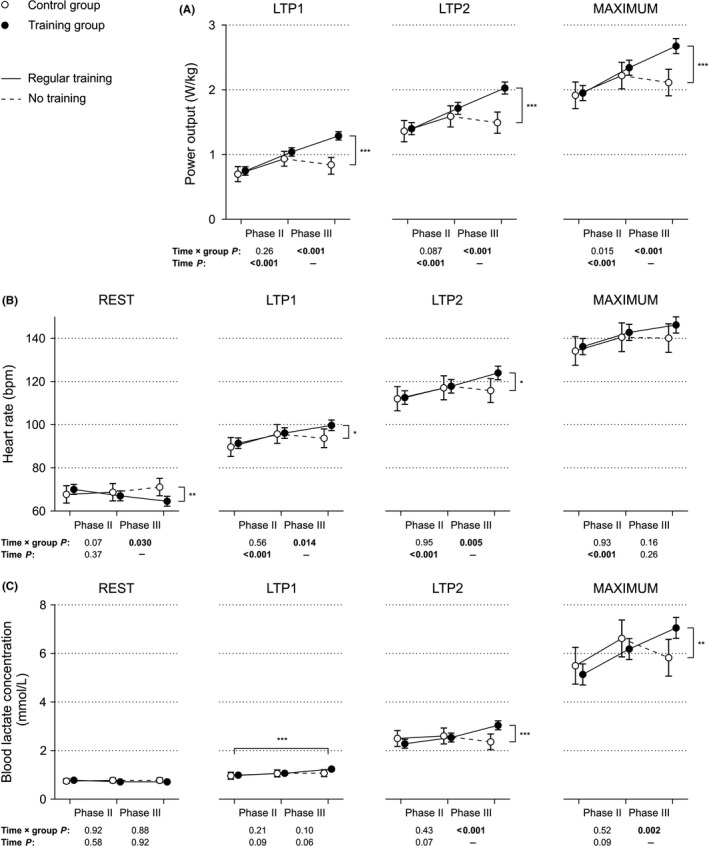
Effects of exercise training during phase II and phase III cardiac rehabilitation on power output, heart rate, and blood lactate concentration. A, Power output at lactate turn points 1 and 2 (LTP1 and LTP2) and maximum values after adjustment for the potential confounders age, smoking status (yes/no), and the use of β‐blockers (yes/no). B, Heart rate at rest, at LTP1, LTP2, and maximum values. Plotted estimated marginal means are adjusted for the potential confounders age, baseline body weight, baseline power output in watts, smoking status (yes/no), and the use of β‐blockers (yes/no). C, Blood lactate concentrations at rest, at LTP1, LTP2, and maximum values. Plotted estimated marginal means are adjusted for the potential confounders age, baseline body weight, baseline power output in watts, smoking status (yes/no), and the use of β‐blockers (yes/no). For all plots, significant time × group interactions indicate that groups developed differently concerning the dependent variable throughout the respective phases of rehabilitation. Significant effects of time indicate that the dependent variable changed in both groups during the respective phase of rehabilitation. *P < 0.05, **P < 0.01, ***P < 0.001, and vertical brackets indicate significant group differences at the end of phase III rehabilitation. The horizontal bracket (blood lactate concentration, LTP1) indicates a difference between time points that applies to both groups. Plotted: Estimated marginal means of both groups with 95% confidence intervals after adjustment for potential confounders. Symbols of both groups at each time point are slightly separated in *x*‐axis direction to avoid overlapping error bars

Cardiorespiratory fitness was indicated by power output at LTP1, LTP2, and at the end of each incremental exercise test in Watt/kg body weight (Figure 3A). During phase II, the power output parameters increased in both groups (time *P* < 0.001 each); during phase III, however, the power output at LTP1, at LTP2, and at maximum developed differently (significant time × group interactions, *P* < 0.001 each). Power output did not differ at the beginning, but at the end of phase III.

Resting HR, HR at LTP1 and LTP2 developed differently over time (significant time × group interactions for the whole study period, *P* < 0.05 each, Figure 3B). During phase II, heart rate at physical rest did not change, whereas HR at LTP1 and LTP2 and maximal heart rate significantly increased in both groups (*P* < 0.001 each, time × group interaction *P* > 0.5 each). In phase III, however, groups differed in changes of heart rate, displaying a decreased resting HR, but increased HR at LTP1 and LTP2 in the TG compared with the CG (time × group interactions < 0.05 each). Of note, there was no evidence of altered maximal HR after phase III in both groups.

Training throughout phase II was not associated with any changes in blood lactate concentrations at rest, at lactate turn points, or at maximum (Figure 3C). Concerning blood lactate concentrations at LTP1, a slight, yet significant increase from baseline to end of phase III by 0.17 mmol/L was observed. During phase III, blood lactate concentrations at LTP2 and at maximum developed differently in both groups.

As an additional exploratory analysis, the power output differences between LTP1 and LTP2, and between maximum and LTP2 were calculated and tested for a time × group interaction (Appendix S1B). Significant improvements elicited by exercise training occurred below LTP1 exclusively. The relative performance at LTP1 and LTP2, as measured as percentage of maximal power output, also changed throughout both phases (Appendix S1C), with higher percentages after phase III in TG patients. BMI did not change differently between groups and overall decreased by 0.27 units throughout phase II (*P* = 0.16, Appendix S1D).

#### 3.2.3 Relationship between changes in cardiorespiratory fitness and changes in *K*_HR_

**Figure 4:**
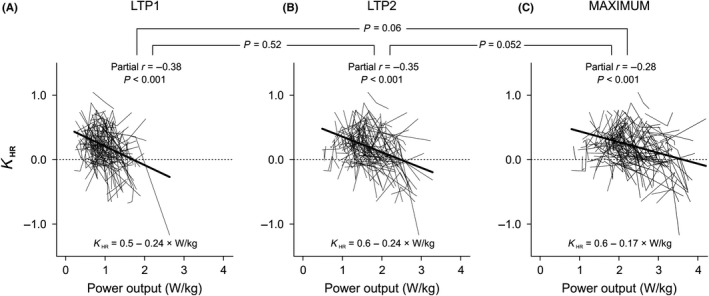
Relationship between intra‐individual changes in cardiorespiratory fitness and changes in the degree of HRPC deflection. Each thin line represents data of a single patient and includes data of three time points. Thick lines are regression lines, with corresponding equations below. Partial correlation coefficients indicate the strength of the association between changes in power output and *K*_HR_. A, Relationship between changes in power output at LTP1 and changes in *K*_HR_. B, Relationship between changes in power output at LTP2 and changes in *K*_HR_. C, Relationship changes in maximal power output and changes in *K*_HR_. Horizontal brackets and *P*‐values refer to the hypothesis that plotted correlations differ concerning their strength (ie, partial *r*)

Partial correlation coefficients between parameters of cardiorespiratory fitness and *K*_HR_ showed negative correlations between changes in power output (at LTP1, at LTP2, and at maximum) and *K*_HR_ (partial *r* = −0.38, −0.35, −0.28, *P* < 0.001 each, Figure 4). For each W/kg body weight power output increase at LTP1, at LTP2, and at maximum that a patient achieves, the estimated associated *K*_HR_ changes were −0.24, −0.24, and −0.17 units. These correlation coefficients did not differ from each other (*P* > 0.05 each).

## 4 DISCUSSION

The main finding of this observational cohort study is that long‐term exercise‐based cardiac rehabilitation was associated with changes in the degree of deflection of the HRPC that can be interpreted as leading toward normalization. Further, long‐term cardiac rehabilitation with exercise training improved cardiorespiratory fitness, and greater improvements of fitness were associated with more pronounced changes of HRPC deflection in the direction of normalization.

The major limitation of this study arises from its retrospective nature. Unknown systematic differences between the two groups, and thus bias, cannot be excluded. For the establishment of a causal effect, a randomized controlled trial would be necessary; however, randomizing patients to a control group without exercise training must be considered unethical, as these subjects would be deprived of the established beneficial effects of rehabilitation on cardiovascular mortality.^2^ In addition, changes in medication that were not included in the statistical model due to limited sample size might have influenced the results. A further limitation is that LVEF was not measured, and it can only be speculated that training in this study was associated with an improvement of myocardial function.

### 4.1 Effects of exercise training on HRPC deflection

Previous studies have shown that the HRPC of young, healthy individuals is usually characterized by a flattening at higher exercise intensities in terms of a downward‐deflected HRPC.^14^ However, in individuals who suffered a myocardial infarction, an upward deflection can frequently be observed.^17^ This upward deflection might be suggestive of a reduced left ventricular function, as the degree of upward deflection, indicated by higher positive values of *K*_HR_, was found to be negatively correlated with left ventricular function during exercise.^17^ However, such a correlation can also be observed in healthy individuals, although reductions in LVEF are within a physiological range.^15^, ^16^ Considering patient characteristics in the study cohort, the mean *K*_HR _was also significantly above zero before rehabilitation, indicating an upward deflection of HRPC, which is in line with previous research.^17^ Additionally, previous work suggests that a percutaneous coronary angioplasty might affect HRPC and LVEF during exercise.^27^ As no such intervention was carried out during the training period, confounding by this variable can be excluded.

Here, we showed for the first time that the HRPC upward deflection improved throughout a 1‐year‐long cardiac rehabilitation, but not within the 6 weeks of phase II. Notably, the 95% confidence interval of mean *K*_HR_ values measured in TG after phase III included zero. Thus, HRPCs after phase II and phase III training can be regarded as linear on average. However, as the downward deflection usually observed in young healthy individuals^14^ was not achieved after 1 year of rehabilitation, we interpreted the observed changes in the HRPC deflection as leading toward normalization.

Additional analyses did neither result in a significant confounding effect of β‐blocker usage, ADP receptor antagonists, statins, ACE inhibitors, nor of diabetes. However, especially the statistical test for diabetes has limited statistical power due to the low number of patients with diabetes in our cohort. The mean *K*_HR_ differences (Appendix S1E) between patients with diabetes and non‐diabetes seem pronounced, and even the treatment effect might be limited. This would be in line with a recent observational study showing that in a different diabetic population (type 1), higher HbA1c levels were associated with more upward‐deflected HRPCs.^18^ Diabetes‐specific training effects on HRPC need to be addressed in the future.

Mechanistically, it might be speculated that the vegetative nervous system is involved in the observed changes of *K*_HR_. Previous studies showed that in healthy individuals, blocking the parasympathetic nervous system by a single dose of atropine caused originally downward‐deflected HRPCs to be more linear or even upward‐deflected, and previously upward‐deflected HRPCs to be even more upward‐deflected.^22^ In contrast, blocking β1‐adrenoceptors by a single dose of bisoprolol exclusively had an effect on originally downward‐deflected HRPCs, causing them to be less downward‐deflected or even upward‐deflected.^24^, ^28^ Notably, these results were obtained in healthy individuals using a single dose of medication and thus do not necessarily apply to our patient cohort, where β‐blockers were taken chronically by some patients. Further, as chronic exercise expands plasma volume, it might be speculated that an increase in blood flow returning from the periphery during acute exercise affects preload, ventricular filling, heart rate, and thus *K*_HR_.

### 4.2 Effects of exercise training on parameters of cardiorespiratory fitness

Changes in power output and resting heart rate can be interpreted as adaptation to endurance training. The effects of a 1‐year‐long exercise training compared with training for 6 weeks demonstrate the importance of long‐term rehabilitation, as suggested by previous studies.^29^, ^30^, ^31^

In the exploratory analysis of maximal power output, an interesting secondary aspect was that output improvements could mainly be attributed to intensities below the first lactate turn point, although training intensity exceeded it. According to a prior study,^32^ this would indicate that the most part of fitness gain was attributable to improvements in local muscular aerobic metabolism. It is tempting to speculate that other forms of exercise training, as high‐intensity interval training, may additionally improve maximal power output by increasing the difference between LTP1 and LTP2, as well as LTP2 and maximal power output, that is anaerobic metabolism. However, this needs to be addressed by future studies.

### 4.3 Relationship between changes in cardiorespiratory fitness and changes in *K*_HR_

Lower *K*_HR_ values reflect improved left ventricular function,^17^ which represents a prerequisite for improved physical performance. As expected, we found that more pronounced increases in cardiorespiratory fitness throughout rehabilitation were associated with a stronger decline of the HRPC upward deflection. Previous research showed that higher levels of habitual physical activity are associated with a better left ventricular function, as measured by a higher LVEF upon maximal exercise.^33^ In synopsis, the facts that *K*_HR _is related to ventricular function^17^ and that *K*_HR_ changed in the present study might be indicative of altered LVEF in our patient cohort. However, it needs to be pointed out that results of previous interventional studies concerning this matter were variable, probably due to heterogeneity in patient characteristics, duration of rehabilitation, and training protocols.^34^

Our findings have important implications for the prescription of exercise intensities based on target heart rate in patients undergoing cardiac rehabilitation. Since the percentage of maximal heart rate as target heart rate for a given training intensity heavily depends on the degree of HRPC deflection,^19^ we suggest that adequate target heart rate should frequently be assessed in patients undergoing cardiac rehabilitation. Otherwise, using the same target heart rate for intensity monitoring despite changes in HRPC deflection might lead to considerable deviations from target intensity. This might cause absence of desired training effects in the case where intensities are too low^9^, ^13^ or, even worse, pose a risk for patients in cases where intensities are too high.^10^ Based on our data, we suggest that a fixed percentage of the maximal heart rate or the heart rate reserve should not, or at least with the utmost caution, be used to prescribe exercise intensity in clinical practice. Rather, suggested target heart rates should be based on thorough exercise testing. Further, as HRPCs change over time, exercise tests should be repeated regularly, and patients should be advised to immediately report any changes in perceived exertion at a given heart rate. Another way to address the problem of altered HRPC deflection would be to prescribe watts (or speed for walking/jogging on treadmills) given the technical feasibility.

### 4.4 Generalizability and limitations

Our findings can be generalized to male patients in cardiac rehabilitation and do not necessarily apply to other populations. Importantly, this issue should be addressed in women as well. Only 23% of all patients of the CARDIOMED rehabilitation center are female, which is in line with previously published data regarding twelve European countries.^35^ Including women in this analysis would have increased variance and thus sample size; wherefore, we confined this study to males. Another limitation is that it is unclear if healthy individuals with a linear or downward‐deflected HRPC respond to exercise training with any changes in HRPC deflection alongside the increase in cardiorespiratory fitness.

Further, our results should be interpreted with caution due to the observational study design. We addressed the issue of confounding by β‐blockers statistically and found no evidence for confounding, which, however, does not completely exclude this possibility. In addition, our data were not sufficient to statistically account for different β‐blocker types and dosages, and the respective changes within patients. Thus, these variables are still to be considered potential confounders.

## 5 PERSPECTIVE

Exercise‐based cardiac rehabilitation over 1 year was associated with a partial normalization of the HRPC deflection and a substantial fitness gain. This normalization was not observed after 6 weeks, which suggests that long‐term cardiac rehabilitation entails additional benefits compared with shorter periods. The partial HRPC normalization should be considered when prescribing exercise intensities by means of a target heart rate and might be indicative of improved cardiac function.

## CONFLICT OF INTEREST

The authors declare that there is no conflict of interest.

## AUTHORS' CONTRIBUTIONS

All authors contributed to conception or design. SH analyzed and interpreted the data. MSL, MH, and HO acquired and interpreted data. IV interpreted data. HG contributed to data acquisition. RP acquired, analyzed, and interpreted data. All authors drafted and critically revised the manuscript, gave final approval, and agree to be accountable for all aspects of the work ensuring integrity and accuracy.
