# Supplementary material for: Exercise‐based cardiac rehabilitation is associated with a normalization of the heart rate performance curve deflection
Source: Scand J Med Sci Sports. 2019 May 28;29(9):1364–74. doi: 10.1111/sms.13462 (PMC6852149; doi:10.1111/sms.13462)
Supplement: Supplementary file 1 [file SMS-29-1364-s001.pdf]

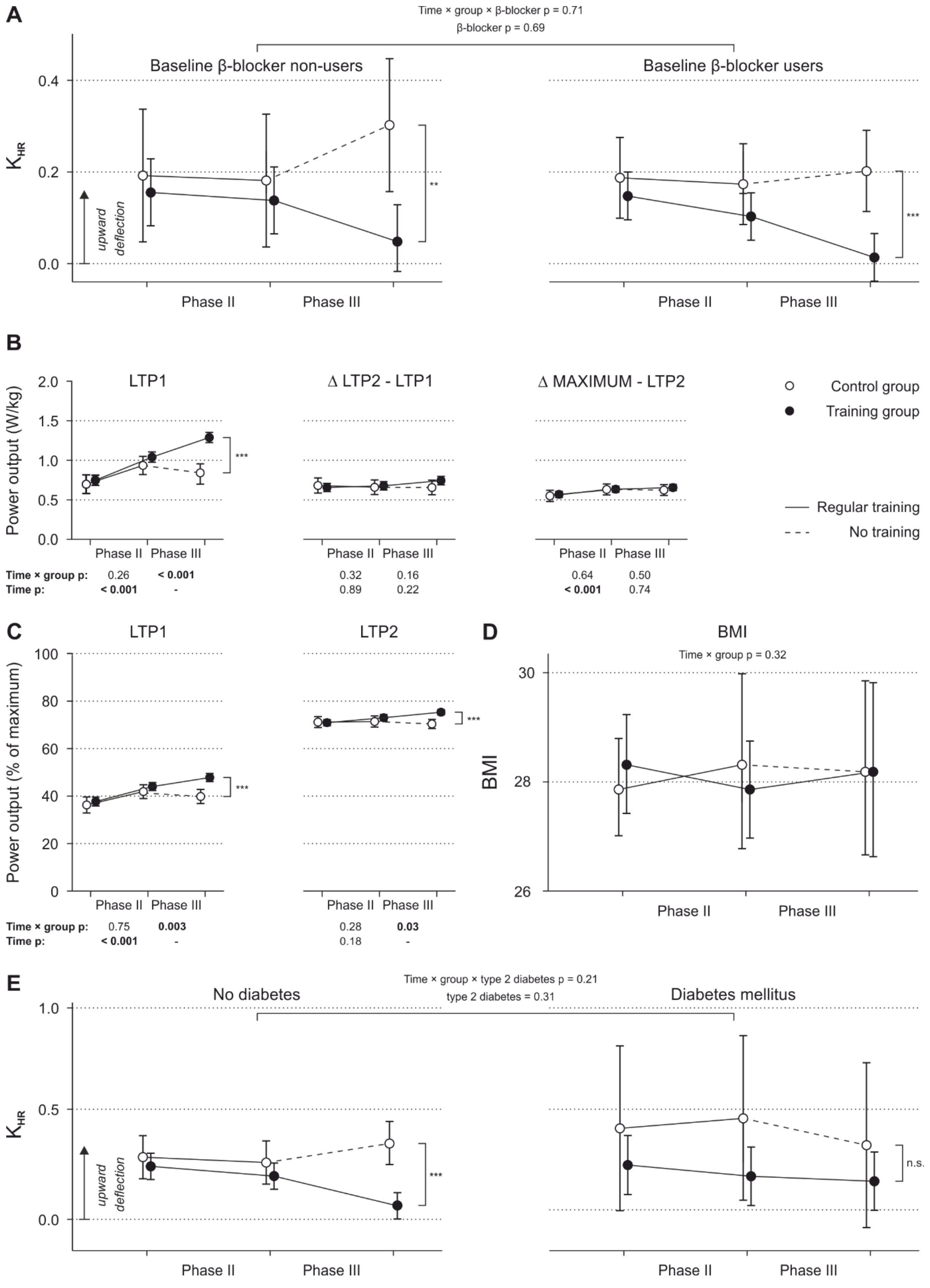

Supplemental digital content:

A: K_{HR}-change over time plotted separately for patients who were taking β-blockers at the beginning of phase II (right, n = 89) and those who were not (left, n = 33). The non-significant

Time × group × β-blocker interaction indicates that there is no evidence for a confounding effect of baseline β-blocker intake on the primary statistical analysis. **B:** Effects of exercise training during Phase II and Phase III cardiac rehabilitation on power output in the range below LTP1 (corresponding to LTP1, left), in the range between LTP1 and LTP2 (corresponding to Δ LTP2 - LTP1, center), and in the range above LTP2 (corresponding to Δ MAXIMUM - LTP2, right). Note that the largest part of training induced alterations occurred below LTP1. The left part of this figure is already shown in Fig. 3 and is plotted for comparability with Δ LTP2 – LTP1 and Δ MAXIMUM – LTP2. **C:** Percentage of maximal power output achieved at LTP1 and LTP2. **D:** Change of BMI over time in both groups. **E:** K_{HR}-change over time plotted separately for patients with (right, n = 25) and without (left n = 107)) diabetes. The non-significant Time × group × type 2 diabetes interaction indicates that there is no evidence for a confounding effect of type 2 diabetes intake on the primary statistical analysis. The non-significant type 2 diabetes-p-value indicated absence of evidence that diabetic patients have higher or lower K_{HR}-values.

Significant time × group interactions indicate that groups developed differently concerning the dependent variable throughout respective phase of rehabilitation. The significant effect of time in Phase II indicates that the power output was increased after Phase II, which applies to both groups. ** $p < 0.01$, *** $p < 0.001$ and the vertical brackets indicate significant group differences at the end of Phase III rehabilitation. Symbols show estimated marginal means of both groups with 95% confidence intervals after adjustment for potential confounders. Symbols of both groups at each time point are slightly separated in x-axis direction to avoid overlapping error bars.
